# Constitutive MAP Kinase Activation in Hematopoietic Stem Cells Induces a Myeloproliferative Disorder

**DOI:** 10.1371/journal.pone.0028350

**Published:** 2011-12-02

**Authors:** Eva Chung, Chia-Lin Hsu, Motonari Kondo

**Affiliations:** 1 Department of Immunology, Duke University Medical Center, Durham, North Carolina, United States of America; 2 Department of Immunology, Toho University School of Medicine, Tokyo, Japan; University of Sao Paulo - USP, Brazil

## Abstract

Myelodysplastic syndromes/myeloproliferative neoplasms (MDS/MPNs) are a group of myeloid neoplasms in which abnormal activation of the Ras signaling pathway is commonly observed. The PI3K/Akt pathway is a known target of Ras; however, activation of the PI3K/Akt pathway has been shown to lead to neoplastic transformation of not only myeloid but also lymphoid cells, suggesting that pathways other than the PI3K/Akt pathway should play a central role in pathogenesis of Ras-mediated MDS/MPN. The MEK/ERK pathway is another downstream target of Ras, which is involved in regulation of cell survival and proliferation. However, the role of the MEK/ERK pathway in the pathogenesis of MDS/MPN remains unclear. Here, we show that introduction of a constitutively activated form of MEK into hematopoietic stem cells (HSCs) causes hematopoietic neoplasms that are limited to MDS/MPNs, despite the multipotent differentiation potential of HSCs. Active MEK-mediated MDS/MPNs are lethal, but are not considered a frank leukemia because it cannot be transplanted into naïve animals. However, transplantation of MDS/MPNs co-expressing active MEK and an anti-apoptotic molecule, Bcl-2, results in T-cell acute lymphocytic leukemia (T-ALL), suggesting that longevity of cells may impact transplantability and alter disease phenotype. Our results clearly demonstrate the proto-oncogenic property of the MEK/ERK pathway in hematopoietic cells, which manifest in MDS/MPN development.

## Introduction

Hematopoietic cells are divided into two groups: lymphoid and myeloid lineages [1]. Leukemia is caused by neoplastic transformation of hematopoietic cells. It is estimated that each year 5.6 and 5.8 out of 100,000 people are diagnosed with lymphoid and myeloid leukemias, respectively [2].

Myeloid neoplasms are sub-divided by the World Health Organization (WHO) into four sub-groups based on morphology, immunophenotype, genetics, and clinical features, which include: acute myeloid leukemia (AML), myelodysplastic syndrome (MDS), myeloproliferative neoplasm (MPN), and a mixed MDS/MPN disease [3]. AMLs are marked by genetic abnormalities, such as chromosomal translocation, and increased blast counts in the peripheral blood or bone marrow. On the other hand, MDSs are generally viewed as pre-leukemic conditions that feature ineffective hematopoiesis that leads to cytopenia(s) [4]. MPNs are a heterogenous collection of diseases that includes leukemic and pre-leukemic conditions marked by increased proliferation of myeloid cells and effective maturation of neoplastic cells [5]. Lastly, the mixed MDS/MPN category, which was added in 2001, was specifically created to accommodate diseases such as juvenile myelomonocytic leukemia (JMML) and chronic myelomonocytic leukemia (CMML) [6]. This modification in the classification scheme was necessary since these diseases did not belong solely in the MDS or MPN category because many cases had both dysplastic and proliferative features [4], [6].

MDS/MPNs are a product of inappropriate activation or inactivation of molecules induced by genetic mutations [7], [8]. As a result, signaling pathways that are normally involved in growth factor receptor signaling are constitutively activated [8], [9]. For example, activation of Ras, a membrane proximal signaling molecule and proto-oncogene is frequently observed in various types of malignant cells generated by neoplastic transformation. In fact, mutations that result in activation of the Ras gene are found in 30% of human cancer cases including leukemia [10], [11]. Moreover, clinical findings also show that Ras mutations are observed in 20–60% of CMML cases and 20–25% of JMML cases [8].

However, the role of Ras in transformation events is unclear because it is located upstream of several pathways, including MEK/ERK, PI3K/Akt, and Ral-GEF [12]. Various mouse models have been generated to further dissect each downstream pathway in order to determine its contribution to malignant transformation of hematopoietic cells. For example, mouse models that feature expression of oncogenic K-Ras, either conferred by a point mutation or ablation of negative regulators such as NF1, result in MPNs that closely resemble human JMML [13], [14], [15], [16], [17].

Activation of the PI3K pathway can be achieved either through mutations that result in constitutive activation of Akt or ablation of PTEN, a negative regulator of Akt [18], [19]. Regardless of the mode of activation, neoplasms that result from activation of the PI3K/Akt pathway begin as pre-leukemic myeloid diseases, but rapidly convert to a lymphoid disease [20], [21], [22], [23]. For example, only 10% of mice that expressed activated Akt in the bone marrow developed AML, while 65% developed an MDS that quickly progressed to T lymphoma [20]. In mice where PTEN expression was ablated, 74% of mice developed T-acute lymphoblastic leukemia (T-ALL) while 26% developed a mixed AML/T-ALL phenotype [21], [22], [23]. On the other hand, activation of K-Ras primarily results in a myeloid disease. The heterogeneity in disease phenotype and latency strongly suggest that PI3K/Akt signaling may not be the sole mediator of oncogenic K-Ras signaling and other downstream pathways such as the MEK/ERK pathway may be involved.

MEK/ERK are dual serine/threonine specific kinases that are part of the classical mitogen-activated protein kinase pathway (MAP kinase) [24]. Of the five MAP kinase pathways, the MEK/ERK pathway is predominantly activated by a variety of extracellular stimuli including growth factors, serum, cytokines, and osmotic stress. This pathway is critically involved in regulating a variety of cellular processes, such as cell growth, migration, proliferation, differentiation, and survival [9], [24].

Although activating MEK/ERK mutations have not been identified in any human samples or cancer cell lines, there is evidence suggesting a link between MEK/ERK and malignant transformation [25]. A constitutively activated form of MEK1 (active MEK hereafter) was generated by deleting a 20 amino acid stretch in the N-terminus that regulates kinase activity and substituting one of the Raf1-dependent regulatory phosphorylation sites (serine 222) with aspartic acid (ΔN3-S222D). Introduction of active MEK resulted in the malignant transformation of NIH3T3 cells [26]. Furthermore, expression of active MEK/ERK is sufficient to relieve growth factor dependency in hematopoietic cell lines [27]. Therefore, activation of the MEK/ERK pathway may play a primary role in malignant transformation of hematopoietic cells, although this issue has not been directly investigated.

In this study, we found that expression of active MEK alone resulted in the development of MDS/MPNs but not other types of hematopoietic malignancies from HSCs. Generation of MDS/MPNs from HSCs expressing active MEK was not affected by enhanced cell survival ability, which was conferred by ectopic expression of Bcl-2, an anti-apoptotic protein. MDS/MPNs induced by expression of active MEK were not transplantable in the presence or absence of ectopic Bcl-2. However, T-ALL with fatal blast crisis was observed in mice transplanted with MDS/MPN cells positive for active MEK and Bcl-2 after a long latency period. These results suggest that expression of active MEK is sufficient for development of myeloid malignancies from HSCs. Moreover, enhanced cell survival of MDS/MPNs could be necessary for the development of blast crisis, which mirrors a similar clinical course observed in human MDS/MPNs. Therefore, we concluded that the MEK/ERK pathway could play a significant role in neoplastic transformation of hematopoietic cells.

## Results

### HSCs expressing active MEK preferentially give rise to mature granulocyte/macrophage (GM) cells *in vivo*, leading to myeloproliferative disorder

**Figure 1:**
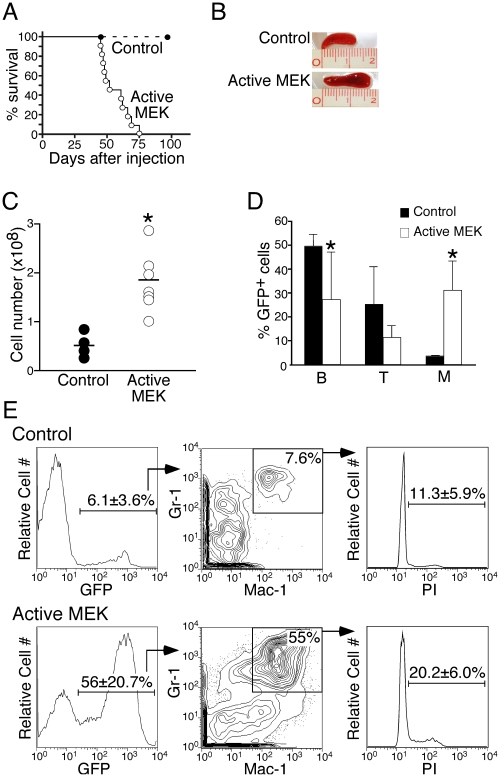
Expression of Active MEK in HSCs led to the development of MPN. (A) A Kaplan-Meier curve illustrating the survival time of mice reconstituted with hematopoietic stem cells (HSCs) infected with control (closed circles, n = 7) or active MEK (open circles, n = 13) retrovirus. Approximately 7,000 retrovirally infected (GFP^+^) HSCs were injected into sublethally irradiated RAG-2^−/−^ recipients. Survival of transplanted mice was monitored for at least 100 days post-injection. (B) Splenomegaly is observed in active MEK mice. (C) Absolute splenocyte counts were determined for mice injected with HSCs transduced with control (filled circle) or active MEK (open circle) retrovirus at the terminal stage. The horizontal bar denotes the mean value for each group of mice. *, *P<0.05* (statistical significance) by Student *t* test. (D) Contribution of MEK^+^ HSCs to different hematopoietic lineages. Bone marrow cells were harvested from transplanted mice at the terminal stage for FACS analysis of the various lineage-specific cell surface markers (B: B220^+^ B cells, T: Thy^+^ T cells, M: Mac-1^+^ myeloid cells). The bar graph summarizes the mean percentage of GFP^+^ donor-derived cells for each cell type for control mice (black bar) and active MEK mice (white bar). Error bars represent the standard deviation from at least 6 mice. *, *P<0.05* (statistical significance) by Student *t* test. (E) GFP^+^ cells in the bone marrow of control (top row, n = 4) or active MEK mice at the terminal stage (bottom row, n = 5) were gated and the percentage of mature myeloid cells (Gr-1^+^ Mac-1^+^) was determined. Gr-1^+^ Mac-1^+^ cells were sorted and cell cycle status was examined using intracellular PI staining. The mean percentage of cells in G_2_/M ± std dev is indicated.

To examine whether activation of the MEK/ERK pathway is sufficient to induce malignant transformation of blood cells, we purified c-Kit^+^Lin^−^Sca-1^+^ (KLS) cells, which are highly enriched for HSCs, from wild type (WT) C57Bl/Ka mice. Using a retroviral transduction system, we introduced active MEK into HSCs and injected them into sublethally irradiated RAG-2^−/−^ mice. The MSCV retroviral vector employed in our model contains an IRES-GFP cassette [28]. Therefore, cells productively infected with retrovirus are positive for GFP expression. Approximately 8 weeks after injection, the mice transplanted with active MEK^+^ HSCs became lethargic, had a hunched back appearance, and experienced weight loss. Moreover, all mice died by 10 weeks post-injection compared to control mice, which remained healthy and free of disease for at least 100 days post-injection (Figure 1A).

We examined mice injected with active MEK^+^ HSCs at the terminal stage as well as mice injected with control virus-infected (GFP^+^) HSCs for comparison. Grossly, splenomegaly of active MEK^+^ mice was obvious (Figure 1B). The cellularity of the spleen was also significantly increased in active MEK^+^ mice compared to control mice (Figure 1C). The marked increase of spleen cells in the presence of active MEK was specifically due to the expansion of Mac-1^+^ myeloid cells at the expense of T and B lymphocyte development (Figure 1D). In addition, the structure of the spleen was disorganized in active MEK^+^ mice (data not shown). Infiltration of cells in the liver and lung of active MEK^+^ mice was also observed (data not shown).

In the bone marrow, average chimerism judged by GFP expression derived from vector control and active MEK mice at the terminal stage was 6.1% and 56%, respectively, suggesting that cells expressing active MEK have more proliferative potential *in vivo* (Figure 1E, left panels). The population of cells that was most prevalent in mice with active MEK was the Gr-1^+^Mac-1^+^ cell population (Figure 1E, center panels), which contains mature myeloid cells.

To distinguish which population was responsible for the expansion of myeloid cells and subsequent MDS/MPN, we purified both c-Kit^+^Sca-1^−^ myeloid progenitors and Gr-1^+^Mac-1^+^ mature myeloid cell populations from moribund active MEK or vector control mice and subjected them to cell cycle analysis. Committed progenitor populations, such as the common myeloid progenitor (CMP), have enhanced cell cycle compared to mature myeloid cells, which are quiescient (Figure S1) [29]. Data obtained from the mature Gr-1^+^Mac-1^+^ population in vector control mice (Figure 1E, right panel) was in good agreement with previously published results showing that only a fraction of mature myeloid cells are cycling [29]. However, active MEK mice displayed a marked increase in the percentage of Gr-1^+^Mac-1^+^ cells in the G_2_/M phase compared to vector control mice (20.2% vs. 11.3%) (Figure 1E, right panels). These data suggest that MEK signaling may specifically affect cell cycle status of mature GM cells rather than more immature myeloid progenitors in the bone marrow. In addition, neither self-renewal potential nor cell longevity of HSCs was influenced by active MEK (Figures S2 and S3). Therefore, it seems that the increase in the number of myeloid cells is due to enhanced proliferation potential of mature GM cells rather than the cells at the progenitor and HSC stage in active MEK^+^ mice.

**Figure 2:**
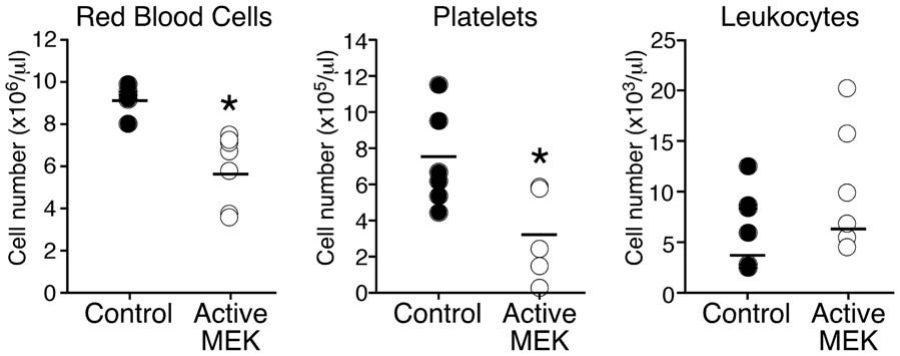
Dysfunctional hematopoiesis in terminally ill active MEK mice. Differential cell counting of blood was performed on peripheral blood harvested from control mice (filled circle, n = 6) and active MEK mice (empty circle, n = 6). Absolute cell counts were determined using a HemaVet machine. The horizontal bar denotes the mean value of each group. *, *P<0.05* (statistical significance) by Student *t* test.

**Figure 3:**
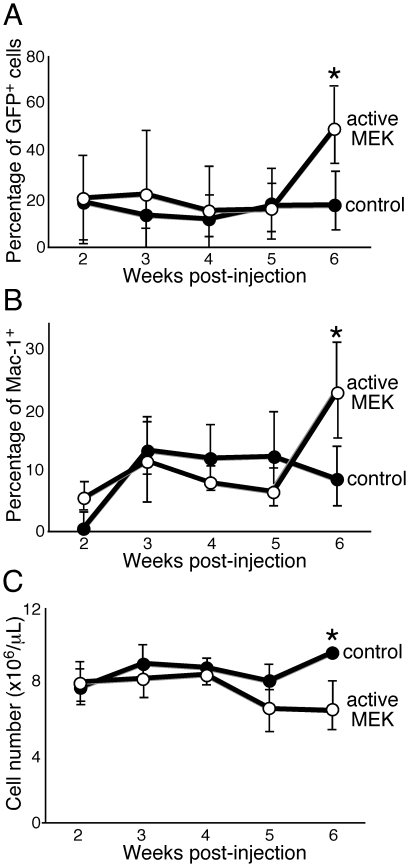
*In vivo* time course analysis of peripheral blood. (A, B) Peripheral blood was collected from vector control (filled circle, n = 3) or active MEK mice (empty circle, n = 5) two weeks post-injection and then weekly, thereafter. Cells were stained with antibodies and subjected to FACS analysis. Cells were pre-gated on GFP^+^ cells and dead cells were excluded from the analysis by PI staining. (C) Differential cell counting was performed on peripheral blood harvested from control mice (filled circle) and active MEK mice (empty circle) using a HemaVet machine to assess red blood cell number. Data shown are the mean values from all mice in each group. Error bars represent the standard deviation from each group of mice. *, *P<0.05* (statistical significance) by Student *t* test.

Next, we performed differential cell counting of the peripheral blood from active MEK^+^ mice at the terminal stage and control mice and found that red blood cell and platelet numbers in active MEK^+^ mice were significantly reduced while a slight increase in leukocytes was observed (Figure 2). We further examined the peripheral blood from the reconstituted mice at different time points following injection of active MEK^+^ and control HSCs. We ended this analysis at 6 weeks post-injection because the mice started to die after this time point. We found that the percentage of GFP^+^ cells in active MEK mice was comparable to vector control mice until week five, when GFP^+^ cells in active MEK mice underwent a large expansion, which also mirrored the expansion of Mac-1^+^ cells (Figures 3A and 3B). Reduction in the number of red blood cells was observed starting at 5 weeks post-injectioin and became significant at week 6 (Figure 3C). Although other studies have suggested that activation of the MEK/ERK pathway may have a negative impact on erythroid differentiation [30], [31], our data suggest that active MEK expression in HSCs does not directly suppress red blood cell production since mice do not develop anemia until later time points (Figure 3C). These results demonstrate that the cause of death of active MEK^+^ mice might be anemia, which appears to be an indirect consequence of the massive expansion of GM cells, and is a common end-stage phenotype observed in myeloid neoplasms.

Dysfunctional hematopoiesis that results in anemia, thrombocytopenia, and decreased lymphocytes as shown above in active MEK^+^ mice at the terminal stage is characteristic of MDS [4], [32]. On the other hand, myeloid cells are able to undergo full and effective maturation. However, the hyperproliferation of mature myeloid cells is indicative of MPNs [4], [32]. MPNs are also marked by leukocytosis, which was observed to a moderate degree in our model (Figure 2). Splenomegly and hepatomegly, two other common signs of MPNs, were also observed in our mice. Taken together, these data suggest that expression of active MEK in HSCs resulted in a mixed MDS/MPN disease, which resembles human JMML and CMML [4], [32]. Therefore, we denote active MEK-induced myeloid disorder reported here as MDS/MPN hereafter.

MDS/MPN includes various classes of myeloid malignancies, some of which are classified as pre-leukemic [7], [33]. A hallmark of leukemia is the ability to recapitulate disease in a naïve animal following transplantation [34]. To determine the transplantability of the disease, we harvested unfractionated bone marrow or spleen cells from individual moribund active MEK^+^ mice and injected them into multiple naïve, sublethally irradiated RAG-2^−/−^ mice. None of the mice, with the exception of one mouse, developed any type of malignancy (data not shown). Therefore, active MEK-induced MDS/MPNs are most likely at the pre-leukemic stage.

### Enhanced cell survival alters the course of hematological malignancies induced by active MEK

Previous reports show that enhanced cell survival conferred by retroviral introduction of activated Akt into bone marrow cells or ablation of PTEN expression in mice results primarily in the development of non-lethal myeloid diseases that progress to a lethal lymphoid disease [20], [21], [22], [23]. To determine whether cell survival would alter disease phenotype in our model, we co-expressed active MEK and Bcl-2, an anti-apoptotic factor. For this purpose, we used a mouse line that expresses the human Bcl-2 transgene and is driven by the MHC class I promoter. All hematopoietic cells in this transgenic mouse express Bcl-2 and are less sensitive to extracellular stimuli that would normally trigger apoptosis such as X-ray irradiation [35], [36].

**Figure 4:**
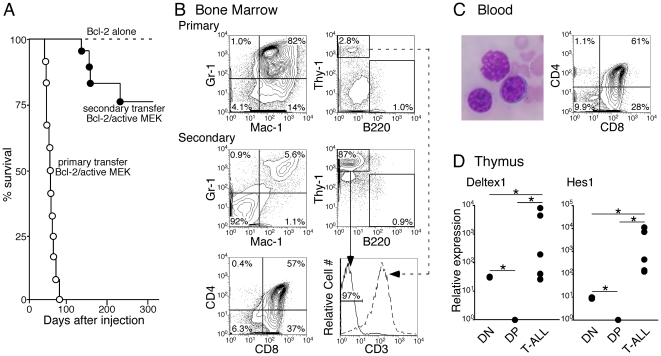
Transplantation of Bcl-2/active MEK^+^-induced MPNs results in greater frequency of T-ALL. (A) A Kaplan-Meier curve illustrating the survival time of primary transplanted mice reconstituted with hematopoietic stem cells (HSCs) harvested from a Bcl-2^+^ transgenic mouse infected with control (dotted line, n = 8) or active MEK (open circles, n = 17) retrovirus and Bcl-2/active MEK^+^ secondary transplanted mice (closed circles, n = 18). Approximately 10,000 retrovirally infected (GFP^+^) HSCs were injected into sublethally irradiated RAG-2^−/−^ recipients for primary transplants. Survival of primary transplanted mice was monitored for at least 120 days post-injection. Cells from the bone marrow or spleen of mice with Bcl-2/active MEK^+^-induced MDS/MPN were injected into sublethally irradiated naïve RAG-2^−/−^ recipient mice. Survival of secondary transplanted mice was monitored for at least 365 days post-injection. (B) At the terminal stage, bone marrow was harvested from moribund primary transplanted (top panel) and moribund secondary transplanted (bottom panel) mice. Secondary transplanted mice were generated by harvesting bone marrow or spleen cells from moribund active MEK or Bcl-2/active MEK^+^ mice and transplanted into naïve recipient mice. Cells were stained with various lineage antibodies and subjected to FACS analysis. CD3 expression on Thy-1^+^ T cells was further analyzed in primary (dotted arrow) and secondary (solid arrow) mice. CD4 and CD8 expression in bone marrow was also examined in secondary transplanted mice. All plots are pre-gated on PI^−^GFP^+^ cells. Data is from one representative mouse out of five mice. (C) Histology of peripheral blood from secondary transplanted mice. Peripheral blood smears were stained with Wright-Giemsa stain (top panel). Zoom = 400×. Peripheral blood was also subjected to FACS analysis to examine CD4 and CD8 expression. Cells were pre-gated on PI^−^GFP^+^ cells. One representative smear and FACS plot is shown for five analyzed mice. (D) Quantitative RT-PCR analysis of deltex-1 (left panel) and hes-1 (right panel) in CD4^−^CD8^−^ DN thymocytes (n = 3), CD4^+^CD8^+^ DP thymocytes (n = 3), and GFP^+^ bone marrow cells from mice with T-ALL (n = 5). Relative expression level for each gene is normalized to β-actin expression level and plotted on a bar graph. The mean normalized expression level for each group was calculated and used to determine fold induction above DP cells. Expression level of the gene was arbitrarily set at 1 for DP cells. Fold induction over DP is expressed for each corresponding group. Standard deviation was calculated for each sample assayed in triplicate. *, *P<0.05* (statistical significance) by Student *t* test.

While all recipient mice injected with Bcl-2/active MEK^+^ HSCs became sick, none of the mice injected with HSCs only expressing the Bcl-2 transgene became sick (Figure 4A). Mice injected with Bcl-2/active MEK^+^ HSCs reached the terminal stage slightly earlier than mice injected with WT HSCs expressing active MEK (average time of death was 56.4 and 66.1 days, respectively) (Figures 1A and 4A). However, Bcl-2/active MEK^+^ mice at the terminal stage also developed an MDS/MPN very similar to those observed in active MEK only mice. Our extensive analysis did not reveal any difference in the disease phenotype observed among RAG-2^−/−^ recipient mice transplanted with active MEK^+^ HSCs in the presence or absence of the Bcl-2 transgene (data not shown). These results demonstrate that enhanced cell survival does not alter disease phenotype and has a minimal impact on disease progression originating from HSCs in the presence of active MEK expression.

Next, we investigated the transplantability of MDS/MPNs initiated by active MEK and Bcl-2. As previously mentioned, only one mouse transplanted with active MEK-induced MDS/MPNs developed disease (5.9%). In contrast, significantly more mice transplanted with Bcl-2/active MEK^+^-induced MDS/MPNs became fatally ill (27.8%, p<0.05). Although primary mice died of MDS/MPNs between 6–10 weeks post-injection of active MEK^+^ or Bcl-2/active MEK^+^ HSCs, secondary mice transplanted with Bcl-2/active MEK^+^ MDS/MPNs did not became sick until at least 15 weeks post-injection (Figure 4A).

We further examined the disease phenotype in the secondary transplanted mice with MDS/MPNs induced by Bcl-2 and active MEK at the terminal stage. Different from primary MDS/MPN mice, we did not observe massive expansion of myeloid cells in the secondary transplanted mice. Rather, the mice had enlarged thymi and Thy-1^+^ T cells became dominant in the bone marrow, spleen, and peripheral blood of the mice (Figures 4B and 4C). A majority of these T cells expressed both CD4 and CD8, suggesting that expanded T cells in the mouse had an immature T cell phenotype similar to the CD4^+^CD8^+^ double positive (DP) population, which is normally observed in the thymus but not in the periphery (Figures 4B and 4C). In accordance with their immature phenotype, T cells from bone marrow and spleen in the secondary transplanted mice were CD3^−^ (Figure 4B). Moreover, peripheral blood smears obtained from these mice show the presence of blast cells in the peripheral blood (Figure 4C). Taken together, these data suggest that the secondary transplanted mice developed T-ALL.

### Notch pathway is exclusively activated in mice with T-ALL

Greater than 50% of human T-ALL cases have been found to harbor mutations that result in aberrant activation of the Notch pathway, which may be necessary for the generation and/or maintenance of disease [37]. Moreover, certain Notch mutations have been shown to enhance the malignancy of tumors generated from introduction of active K-Ras [38].

To determine whether activation of the Notch pathway is involved in T-ALL generation in the secondary transplanted mice that received bone marrow or spleen cells harvested from MDS/MPN mice co-expressing active MEK and Bcl-2, we examined expression of Notch target genes as performed previously [39]. As shown in Figure 4D, T-ALL cells expressed significantly higher levels of the Notch target genes Hes1 and Deltex1 when compared to phenotypically similar WT DP thymocytes, which only express basal levels of these genes. Among hematopoietic cells, CD4^−^CD8^−^ double negative (DN) thymocytes exhibit high levels of Notch signaling [40]. However, the level of Notch signaling observed in our diseased animals exceeds even that found in DN thymocytes (Figure 4D), which is in agreement with previous studies showing that the levels of Notch signaling observed in T-ALLs far exceeds those necessary to mediate physiological functions [38]. Therefore, activation of the Notch pathway may be necessary to complete malignant transformation of cells initiated by active MEK.

## Discussion

MEK/ERK transformation potential has been demonstrated in various cell line systems; however, direct proof in primary hematopoietic cells was lacking [27], [41]–[43]. In this report, we provided direct evidence that introduction of active MEK into HSCs is sufficient to give rise to a MDS/MPN. The MDS/MPN we observed has features of both CMML and JMML such as lack of the *BCR/ABL* fusion gene, persistent peripheral blood monocytosis, and non-elevated myeloblast or monoblast counts in the bone marrow and peripheral blood [4], [8].

Additionally, our mice also show signs of anemia and thrombocytopenia, which are characteristic of myeloid dysplasias observed in CMMLs [4], [8]. This myeloid dysplasia was observed only at later time points, which coincided with an increase in the percentage of donor-derived GM cells (Figure 3A). These GFP^+^ cells were mainly Mac-1^+^ (Figure 3B), suggesting that active MEK signaling does not directly effect erythropoiesis and platelet production (Figures 2 and 3C). The Gr-1^+^Mac-1^+^ GM population of active MEK mice was found to be cycling more than vector control mice (Figure 1E). Therefore, anemia and thrombocytopenia observed at the terminal stage of active MEK mice could be a consequence of enhanced mature myeloid cell proliferation, which could out-compete normal cells for space in the bone marrow resulting in dysfunctional hematopoiesis.

Also, our mice display organomegaly and infiltration of mature myeloid cells into non-hematopoietic organs, which is more often associated with JMML [7], [8]. While a majority of JMML cases have been linked to activated Ras, the etiology of CMML has been found to be more heterogeneous, suggesting that dysregulation of multiple pathways can lead to similar disease phenotypes [8].

The phenotype of active MEK-mediated myeloid disorders observed in our study is similar to oncogenic K-Ras- but not PI3K/Akt-mediated myeloid neoplasms [14], [15], [16], [17]. Additionally, inhibition of MEK signaling in oncogenic K-Ras animals enhances survival and improves myeloid progenitor function [44]. These data suggest that MEK may be the primary mediator of oncogenic K-Ras signaling. Activation of the PI3K/Akt pathway appears to favor development of mixed lineage diseases that begin as MPNs, but rapidly progress to more aggressive diseases, namely T cell lymphomas and T-ALLs, while only a small percentage develop AML [20], [21], [22], [23]. Therefore, MEK/ERK and PI3K share some overlapping functions, but also have distinct roles in neoplastic transformation of hematopoietic cells.

The PI3K/Akt pathway is primarily associated with cell survival; however, it is well-known that sole enhancement of cell survival is not sufficient to cause malignant transformation [45]. For example, translocation events that placed Bcl-2 under the control of the immunoglobulin heavy chain enhancer coupled with dysregulated c-Myc expression resulted in lymphoid disease. However, ectopic expression of Bcl-2 alone is insufficient to promote oncogenesis [46], [47]. These data suggest that dysregulation of the cell cycle and uncontrolled proliferation may be primary transformation events while prolonged cell survival may play a supportive role, which allows susceptible pre-leukemic cells to acquire additional mutations that can lead to blast crisis.

Active MEK-induced MDS/MPN was not transplantable into naïve mice, highlighting the pre-leukemic nature of the disease. These observations led us to ask which cell population was responsible for the generation of active MEK-induced MDS/MPN. We investigated self-renewal potential of active MEK^+^ HSCs by a serial replating assay on methylcellulose medium and found that expression of active MEK does not enhance self-renewal capacity of HSCs (Figure S2). Therefore, it is unlikely that HSC potential is enhanced by active MEK. Rather, the increase in myeloid cells most likely occurred at the mature Gr-1^+^Mac-1^+^ stage rather than at the progenitor stage (Figures 1E and S1). Therefore, the numerous progenies that resulted from enhanced cell cycle status of mature myeloid cells cannot be sustained and may not be able to generate the same disease in the secondary transplanted mice. On the other hand, even if transformed progenitors capable of initiating lymphoid disease were present, we would not be able to detect them in the primary mice transplanted with active MEK^+^ HSCs because the MDS/MPN was lethal and had a relatively short latency period (Figure 1A).

While MEK signaling has been shown to be involved in promoting cell survival, it did not block apoptosis in HSCs cultured in the absence of growth factor (Figure S3). However, ectopic expression of Bcl-2 was able to extend this latency period, which may have allowed multipotent or lymphoid-skewed progenitors to persist, increasing the likelihood of acquiring additional mutations. Thus, these conditions may be more favorable for T-ALL development in Bcl-2/active MEK^+^ secondary transplanted mice than secondary transplanted active MEK only mice. All T-ALLs, regardless of the source of primary MDS/MPN tumors, were similar. Lymphoblasts in the peripheral blood and cellular infiltration of multiple organs were observed. These lymphoblasts expressed both CD4 and CD8 on their cell surface, but not CD3 (Figures 4B and 4C), which is in good agreement with their immature morphology.

Further analysis showed that T-ALLs had activated Notch signaling, whereas MDS/MPNs did not. Our data is in agreement with models of oncogenic K-Ras where T-ALL development occurs only after a long latency period and genetic aberrations such as activating mutations and chromosomal alterations are observed [16], [17], [48]. These data suggest that enhanced cell survival may have contributed to the acquisition of additional mutations that promote leukemogenesis.

Similarly, active inhibition of apoptosis has been suggested to be necessary for development of lymphoid neoplasms [49]. Overexpression of the oncogene c-Myc in hematopoietic cells resulted in increased apoptosis and AML development in primary and secondary transplanted mice. Coupling c-Myc and Bcl-2, an anti-apoptotic gene that is able to counteract c-Myc-induced apoptosis, resulted in lymphoid and myeloid diseases in the primary transplant, but only lymphoid disease in the secondary transplant [49]. These data suggest that aberrant expression of oncogenes that promote cell survival may allow lymphoid leukemia initiators to acquire additional oncogenic “hits”. These newly acquired “hits” can cooperate with existing oncogenes, which can significantly affect disease progression and phenotype.

MEK/ERK signaling has been implicated in a variety of cellular functions including proliferation, survival, and differentiation. The results shown in this paper clearly demonstrate that active MEK can function as an oncogene in hematopoietic cells, suggesting that MEK/ERK signaling must be tightly regulated in this cell type. However, we should note that only myeloid disorders were observed in recipient mice injected with HSCs expressing active MEK despite the fact that HSCs have multipotent hematopoietic differentiation potential.

We previously demonstrated that activation of the MEK/ERK pathway promotes granulocyte/macrophage differentiation from HSCs both in vivo and in vitro, most likely through its ability to upregulate C/EBPα, a transcription factor crucial for myeloid cell development that has also been shown to regulate HSC self-renewal [50]–[52]. In addition, ERK can phosphorylate c-Myc, a well-known cell-cycle regulator, protecting the protein from proteasomal degradation [53], [54]. Reciprocal expression of C/EBPα and c-Myc has been observed in monocyte cell lines and they have also been shown to negatively regulate the expression of each other [55]–[57]. This suggests that differentiation and proliferation are mutually exclusive cellular events that do not occur while the other is in progress. MEK signaling fluctuates as HSCs commit to the myeloid lineage and differentiate into mature cells; thus, turning off MEK signaling is important not only to control cellular proliferation, but also to balance HSC output.

Taken together, our findings provide new insight into the novel role of the MEK/ERK pathway in leukemogenesis. Our data suggests that activated MEK can serve as an oncogene in hematopoietic cells because its activation is sufficient to drive development of MDS/MPN. Moreover, enforced expression of Bcl-2 allows transformed cells to acquire additional oncogenic hits, such as activated Notch signaling. It will be interesting to examine the interplay between the MEK/ERK and Notch pathways because both pathways have been shown to be important for differentiation. Our experimental systems are unique and may be helpful in future investigations of T-ALL development stemming from MDS/MPNs.

## Materials and Methods

### Mice

We used 6–12 weeks of age C57Bl/Ka-Thy1.1-Ly5.2, H2K-*BCL-2* transgenic, and RAG-2^−/−^Ly5.2 mice in this study. All mice were maintained under specific pathogen-free conditions at the Duke University Animal Care Facility. All procedures were approved by IACUC at Duke University (permit number, A202-10-08).

### Cytology and complete blood cell counts

Tail vein bleeds were used to collect peripheral blood for weekly analysis of reconstituted animals. Blood was collected in a heparinized tube and differential cell counting was done with a HemaVet 950FS (Drew Scientific Inc.). Peripheral blood was collected as described and smeared onto glass slides and stained with Wright-Giemsa stain to examine cytology.

### Cell sorting and FACS analysis

For cell surface phenotyping, cells were incubated with normal rat IgG (Sigma), followed by fluorescence- or biotin-conjugated antibodies on ice for 20 min. Biotinylated antibodies were further incubated with fluorochrome-conjugated streptavidin after washing with staining medium (Hanks' balanced salt solution (Invitrogen, Carlsbad, CA) with 2% fetal calf serum and 0.02% NaN_3_).

Antibodies used in fluorescence-activated cell sorting (FACS) and analysis are as follows: phycoerythrin (PE) anti-Thy1.2, PE-anti-CD8, PE-anti-Sca-1, PE/Cy5 anti-CD3, PE/Cy5 anti-CD4, PE/Cy5 anti-CD8, PE/Cy5 anti-B220, PE/Cy5 anti-CD19, PE/Cy5 anti-Gr-1, PE/Cy5 anti-Mac-1, PE/Cy5 anti-Ter119, PE/Cy5 anti-Thy1.1, PE/Cy5 anti-Thy1.2, PE/Cy7 anti-Gr-1, allophycocyanin (APC)-anti-Mac-1, APC anti-Thy1.2, APC anti-c-Kit, APC/Cy7 anti-B220, APC/Cy7 anti-Mac-1, APC/Cy7 anti-CD4, biotinylated CD3. All of the above were purchased from eBioscience (San Diego, CA), BioLegend (San Diego, CA), or R&D (Minneapolis, MN). Alexa Fluor 594-anti-Sca-1 was prepared in our laboratory with a standard procedure. Biotin-conjugated antibodies were visualized with PE/Cy7-steptavidin (eBioscience).

FACS sorting and analysis were done on a FACSVantage with DiVa option equipped with a 488 nm argon laser, a 599 nm dye laser, and a 408 nm krypton laser (BD Biosciences Flow Cytometry Systems), which is available in the FACS facility at the Duke University Comprehensive Cancer Center. Data were analyzed with the FlowJo software (Treestar, Ashland, OR). Dead cells that stained positively by propidium iodide were excluded from analyses and sorting.

### Retroviral production and gene transfer

Active MEK cDNA was cloned into the MSCV-IRES-GFP vector. We produced virus using 293T cells, which were maintained in Dulbecco modified Eagle medium (DMEM) (Invitrogen, Carlsbad, CA) supplemented with 10% FBS as described elsewhere [28]. Briefly, 293T cells were transfected with viral constructs along with gag-pol and VSV-G constructs. Viral supernatants were collected for 4 days and concentrated by ultracentrifugation at 5×10^4^ *g* for 4 hrs at 4°C. Virus was aliquoted and stored at −80°C until use.

### Retroviral bone marrow transplantation assay

To purify HSCs (Lin^−^Sca-1^+^c-kit^+^), we harvested bone marrow cells from tibia and femurs from C57Bl/Ka-Thy1.1-Ly5.2 or H2K-*BCL-2* transgenic mice at 6–12 weeks of age. Bones were flushed with ice-cold staining medium (Hanks' balanced salt solution containing 2% FBS and 0.02% NaN_3_) to create a single-cell suspension. Cells were kept on ice for the duration of the procedure, unless noted. Cells were pelleted by centrifugation at 1,500 rpm for 5 min at 4°C. Cells were then treated with ACK lysis buffer for 1 min at 25°C to lyse red blood cells and washed with staining media.

Next, c-Kit^+^ cells were enriched by staining whole bone marrow with anti-CD117/c-Kit microbeads (Miltenyi Biotec, Auburn, CA) and isolating positively labeled cells using the POSSEL_S mode with autoMACS cell separation. Cells were then incubated with rat IgG to block non-specific antibody binding. Cells were then stained with anti-Sca-1, anti-c-Kit, anti-Lineage (CD3, CD4, CD8, B220, Gr-1, Mac-1, Ter119) antibodies for 20 min at 4°C.

HSCs were sorted and cultured at 37°C overnight in X-Vivo 15 (BioWhittaker, Walkersville, MD) supplemented with 10% FBS, stem cell factor (SCF) (50 ng/mL, R&D Systems, Minneapolis, MN), thrombopoietin (10 ng/mL, R&D Systems, Minneapolis, MN), and IL-11 (10 ng/mL, R&D Systems, Minneapolis, MN). Active MEK or control retroviral supernatant was added to the cells the following day and cells were spun at 2,000 rpm at 25°C for 2 hrs. Cells were further cultured at 37°C for 24 hrs to allow for gene expression. Cells were harvested and GFP^+^ cells were sorted, washed with ice-cold staining medium, and resuspended in RPMI1640 (Invitrogen, Carlsbad, CA) with 10% FBS. HSCs (1×10^4^) were injected into the tail vein of sublethally irradiated (400 rad) RAG-2^−/−^ Ly5.2 mice. Eleven active MEK and 7 control mice were analyzed. Also, 13 Bcl-2/active MEK^+^ and 10 control mice were analyzed. Following transplantation, recipient mice were maintained on antibiotic water (sulphamethoxazole and trimethorpim) and evaluated routinely for signs of morbidity, weight loss, hunchback appearance, and splenomegaly. Pre-morbid animals were sacrificed and tissues were harvested and analyzed by flow cytometry and histopathology.

For secondary transplantations, unfractionated bone marrow and spleen cells from individual primary transplanted mice were harvested, washed, and immediately injected into the tail vein of sublethally irradiated (400 rad) RAG-2^−/−^ Ly5.2 mice. Approximately 5×10^5^–1×10^6^ bone marrow or 5×10^6^–10×10^6^ splenocytes harvested from individual primary transplanted mice were injected into multiple recipient mice. Eighteen mice were analyzed for both the active MEK and Bcl-2/active MEK^+^ secondary transplant groups. Following transplantation, recipient mice were maintained on antibiotic water and evaluated routinely for signs of disease as described above.

### Cell cycle analysis

Cell populations were isolated and sorted based on specific markers described in the text as previously described. Cells were washed with staining media, pelleted, and the supernatant was removed. Next, ice-cold 70% ethanol was added drop-wise to the sample while vortexing vigorously. Samples were stored in the dark at 4°C for a minimum of 16 hrs and up to 48 hrs. Cells were pelleted and the ethanol was carefully aspirated from the top. Cells were resuspended in 200 µL of PI buffer (1×PBS with 0.1% glucose containing PI and RNase A) and read on a flow cytometer.

### Gene expression analysis of bone marrow cells

RNA purification and first-strand DNA synthesis were done as previously described [39]. Briefly, cells were lysed using 1 ml of TRIzol reagent (Invitrogen). Total RNA was purified based on the manufacturer's instructions. First-strand cDNA was synthesized with Superscript III RT and random hexamers (Invitrogen). Quantitative PCR was performed in triplicate on MyIQ (Bio-rad). The expression level of the gene of interest was calculated and normalized to Rpl-3 or β-actin. PCR primers are listed below

β-actin-F: 5′-GGGAATGGGTCAGAAGGAT-3′

β-actin-R: 5′-GGGGTGTTGAAGGTCTCAAA-3′

Deltex1-F: 5′-CAGCCGCCTGGGAAGATGGAGTT-3′

Deltex-1-R: 5′-TGGATGCCTGTGGGGATGTCATAGAC-3′

Hes-1-F: 5′-AAAGCCTATCATGGAGAAGACGCG-3′

Hes-1-R: 5′-GGAATGCCGGGAGCTATCTTTCTT-3′

### Methylcellulose culture systems

HSCs were sorted and transduced with retrovirus as described above. After 48 hrs of infection, cells were harvested, stained with KLS antibodies as previously described, and GFP^+^ HSCs cells were sorted. Sorted cells were washed with staining media, cells were resuspended to a concentration of 500 cells/10 µL of IMDM/well, and 500 µL of MethoCult 3534 (StemCell Technologies)/well was added directly to the cells. Tubes were capped and vortexed vigorously. Cells were incubated at 25°C for 20–30 min and then plated in a 24-well plate using a syringe and 18 ½ gauge needle. Samples were plated in quadruplicate. Colonies were enumerated 5–8 days after plating.

Secondary platings were carried out after enumeration. Cells were harvested, washed twice with PBS, and counted. Cells were resuspended to a concentration of 5×10^4^ cells/10 µL IMDM/well, and 500 µL of MethoCult 3534/well was added directly to the cells. Cells were processed as previously described. Samples were plated in quadruplicate. Colonies were enumerated 5–8 days after plating.

### Detection of apoptotic cells

HSCs were sorted and infected with retrovirus as previously described. Following retroviral transduction, cells were harvested and GFP^+^ HSCs were sorted directly into IMDM (Invitrogen, Carlsbad, CA) with 1% BSA. Cells were pelleted, resuspended in IMDM with 1% BSA, and plated. Cells were harvested 6 or 12 hrs later and assayed for apoptosis. Cells were washed with Annexin V binding buffer (BioLegend, San Diego, CA), incubated with Annexin V (BioLegend, San Diego, CA) and 7-AAD antibodies (Invitrogen, Carlsbad, CA) for 15 min on ice, washed twice with Annexin V binding buffer, and analyzed using a flow cytometer.

## Supporting Information

Figure S1 **Cell Cycle analysis of myeloid progenitors.** DNA content of myeloid progenitors (Lin^−^ c-kit^+^) with or without active MEK was assessed by intracellular PI staining.
(TIF)

Figure S2 **Expression of active MEK in HSCs does not enhance self-renewal.** (A–C) HSCs were transduced with retrovirus expressing vector control (black bars) or active MEK (white bars). Following transduction, GFP^+^ cells were sorted, 500 cells/well were plated on methylcellulose media, and colonies were enumerated 5–8 days later. The total colony number is shown in (A). Shape of the representative colony is shown in (B). The avarage number of colony forming cells is shown in (C). (D) The total number of colonies in the secondary culture of colony forming cells in (A–C). NS, *P>0.05* (statistical significance) by Student *t* test.
(TIF)

Figure S3 **Active MEK does not enhance cell survival in HSCs.** Active MEK^+^ and control HSCs were cultured in IMDM with 1%BSA at 37°C for 12 hrs, harvested, washed, stained with AnnexinV and 7-AAD, and read on a flow cytometer. Results shown are representative of three independent experiments.
(TIF)
