# Supplementary figures and images for: Constitutive MAP Kinase Activation in Hematopoietic Stem Cells Induces a Myeloproliferative Disorder
Source: PLoS One. 2011 Dec 2;6(12):e28350. doi: 10.1371/journal.pone.0028350 (PMC3229546; doi:10.1371/journal.pone.0028350)

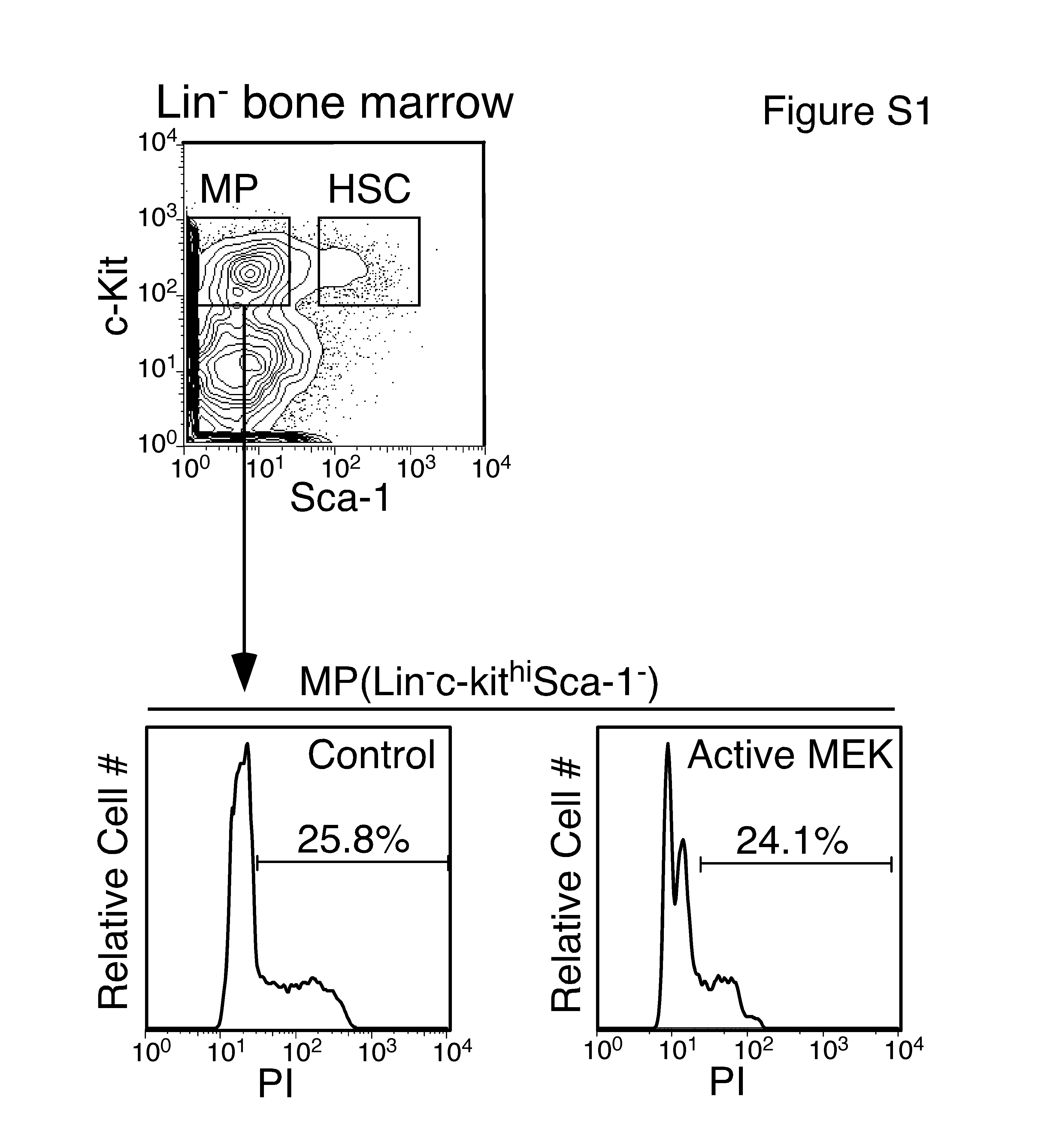

Supplement: Figure S1 — Cell Cycle analysis of myeloid progenitors. DNA content of myeloid progenitors (Lin− c-kit+) with or without active MEK was assessed by intracellular PI staining. (TIF) [file pone.0028350.s001.tif]

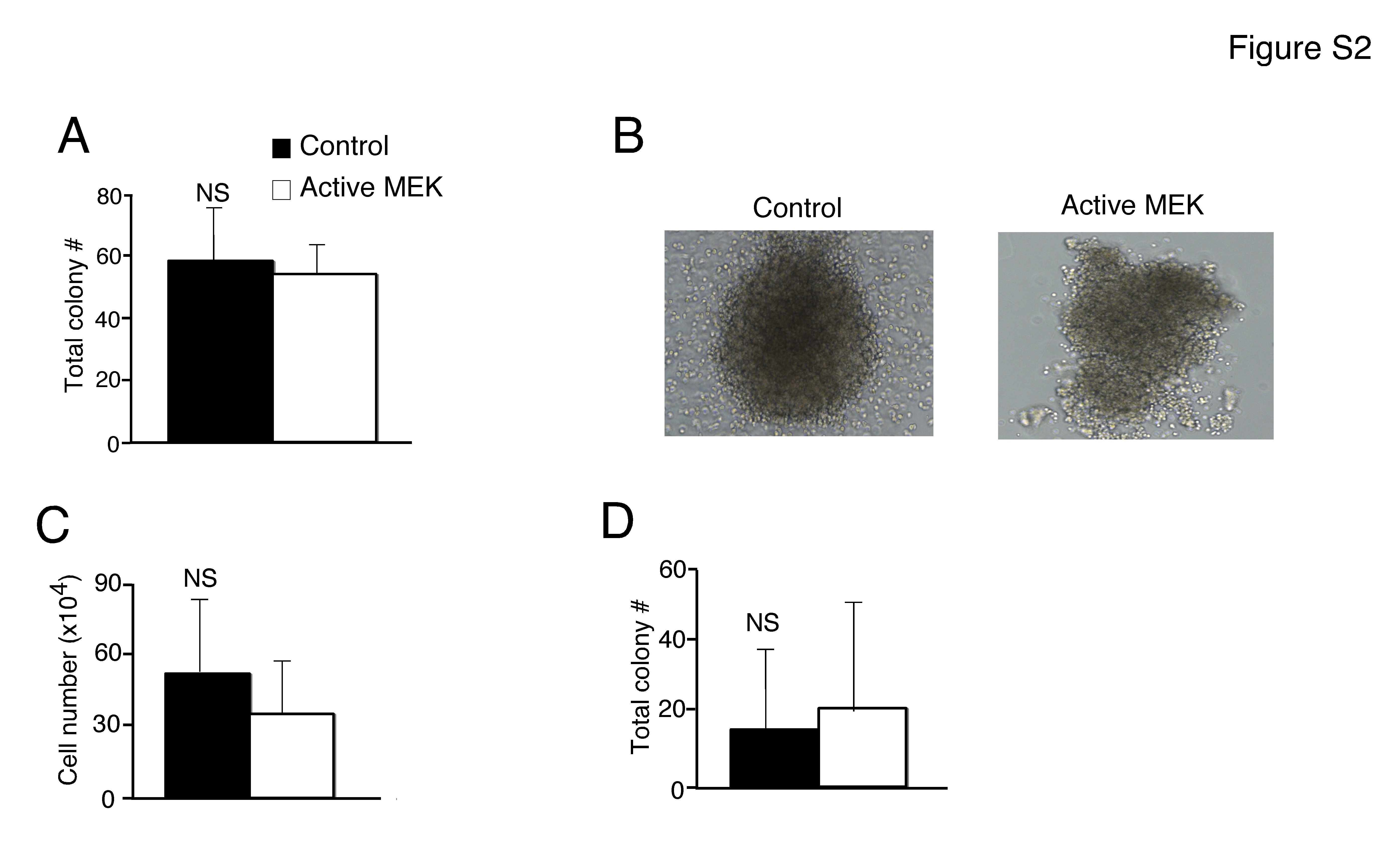

Supplement: Figure S2 — Expression of active MEK in HSCs does not enhance self-renewal. (A–C) HSCs were transduced with retrovirus expressing vector control (black bars) or active MEK (white bars). Following transduction, GFP+ cells were sorted, 500 cells/well were plated on methylcellulose media, and colonies were enumerated 5–8 days later. The total colony number is shown in (A). Shape of the representative colony is shown in (B). The avarage number of colony forming cells is shown in (C). (D) The total number of colonies in the secondary culture of colony forming cells in (A–C). NS, P>0.05 (statistical significance) by Student t test. (TIF) [file pone.0028350.s002.tif]

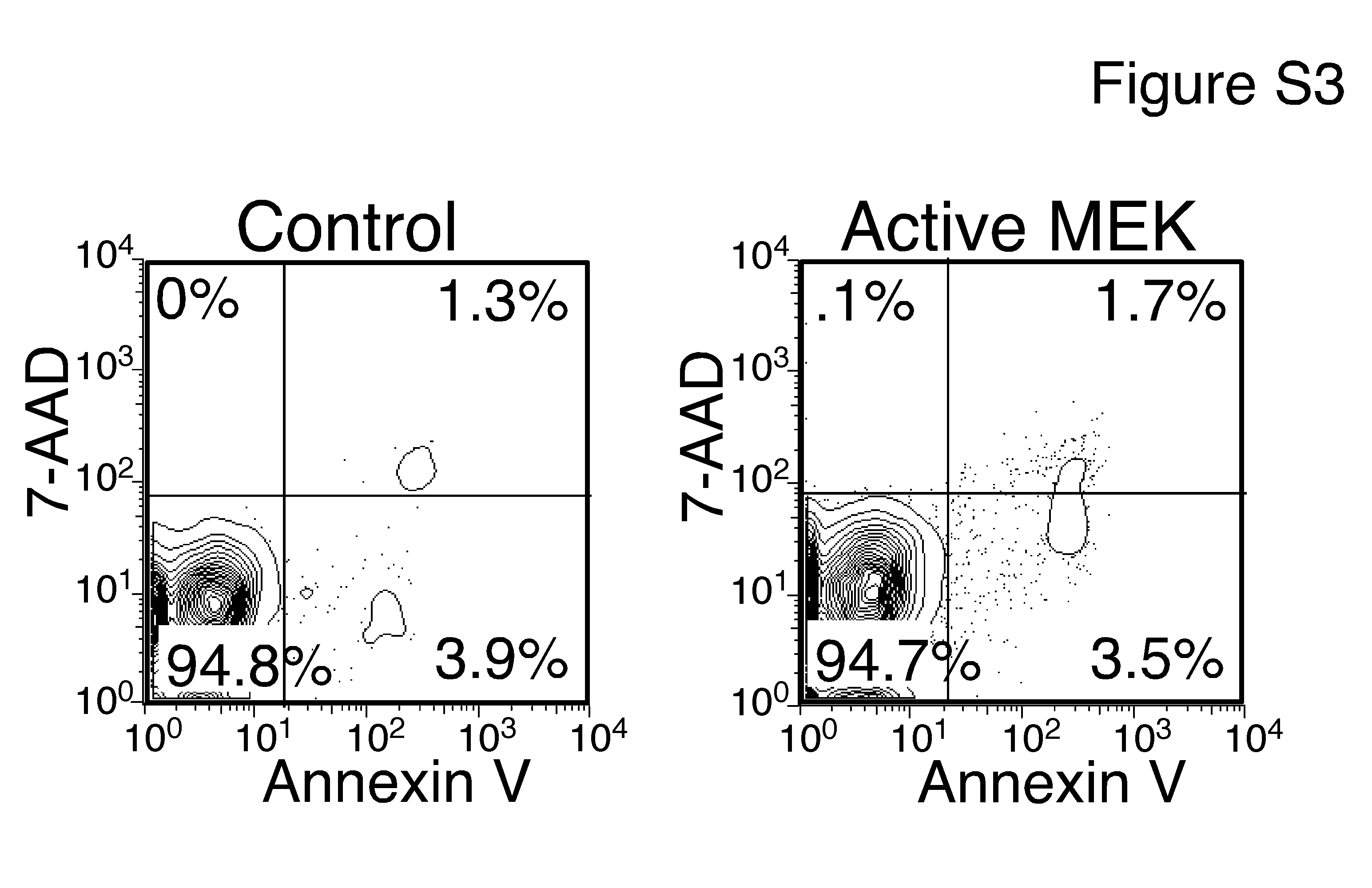

Supplement: Figure S3 — Active MEK does not enhance cell survival in HSCs. Active MEK+ and control HSCs were cultured in IMDM with 1%BSA at 37°C for 12 hrs, harvested, washed, stained with AnnexinV and 7-AAD, and read on a flow cytometer. Results shown are representative of three independent experiments. (TIF) [file pone.0028350.s003.tif]
